# Hypoxic tumour cell-derived exosomal miR-340-5p promotes radioresistance of oesophageal squamous cell carcinoma via KLF10

**DOI:** 10.1186/s13046-021-01834-9

**Published:** 2021-01-23

**Authors:** Fangyu Chen, Bing Xu, Jie Li, Xi Yang, Junjie Gu, Xijuan Yao, Xinchen Sun

**Affiliations:** 1 The First School of Clinical Medicine, Nanjing Medical University, Nanjing, China; 2 Department of Radiation Oncology, The First Affiliated Hospital of Nanjing Medical University, No.300 Guangzhou Road, Nanjing, 210029 China; 3 Department of Radiation Oncology, Fudan University Shanghai Cancer Center, Shanghai, China; 4 Department of Oncology, Shanghai Medical College, Fudan University, Shanghai, China

**Keywords:** Hypoxia, Extracellular vesicles, miRNA, Oesophageal cancer, Radiotherapy

## Abstract

**Background:**

Radiotherapy resistance is a major obstacle in the treatment of oesophageal squamous cell carcinoma (OSCC). Hypoxia is a critical cause of radioresistance. However, the communication between hypoxic cells and aerobic cells via exosomes during the transfer of radiation resistance remains unclear.

**Methods:**

Exo-miR-340-5p levels were analysed by RNA-seq and qRT-PCR. We co-cultured OSCC cells with isolated normoxic and hypoxic exosomes to study their impact on radiosensitivity. We used a specific exo-miR-340-5p mimic and knock-down retrovirus to explore the role of this miRNA in the transfer of radioresistance from hypoxic to normoxic cells. Dual-luciferase reporter and RIP assays were used to verify KLF10 as a putative target of miR-340-5p. Several in vitro assays were conducted and xenograft models were established to investigate the effect of exo-miR-340-5p on OSCC radiosensitivity. The plasma exo-miR-340-5p levels in OSCC patients were analysed to study the clinical value of this parameter.

**Results:**

Hypoxic exosomes alleviated radiation-induced apoptosis and accelerated DNA damage repair. miR-340-5p was highly expressed in hypoxic exosomes and was transferred into normoxic cells, where it induced radioresistance. Overexpression of miR-340-5p in normoxic OSCC cells mimicked the radioresistance of cells co-cultured with hypoxic exosomes. Knockdown of miR-340-5p in hypoxic exosomes reversed the radioresistance effect, indicating that exo-miR-340-5p is critical for hypoxic EV-transferred radioresistance. KLF10 was identified as the direct target of miR-340-5p. Moreover, metformin was found to increase the expression of KLF10 and enhance the radiosensitivity of OSCC. Higher levels of miR-340-5p in the plasma exosomes from OSCC patients are related to a poorer radiotherapy response and prognosis.

**Conclusions:**

Hypoxic tumour cell-derived exosomal miR-340-5p confers radioresistance in OSCC by targeting KLF10/UVRAG, suggesting that miR-340-5p could be a potential biomarker and therapeutic target for the enhancement of radiosensitivity in OSCC. Metformin can increase KLF10 expression, which ameliorates the radioresistance induced by exo-miR-340-5p transfer. Therefore, metformin could be further investigated as a therapeutic option for the treatment of OSCC.

**Supplementary Information:**

The online version contains supplementary material available at 10.1186/s13046-021-01834-9.

## Background

Oesophageal cancer ranks sixth in cancer-associated deaths worldwide. It has been reported that 572,034 oesophageal cancer diagnoses and 508,585 deaths occurred in 2018 [1]. In China, more than 95% of oesophageal cancer patients are diagnosed with oesophageal squamous cell carcinoma (OSCC) [2]. Most patients are diagnosed with locally advanced OSCC. For these patients, radiotherapy is an essential strategy [3]. Despite advances in radiotherapy, the prognosis of OSCC has been disappointing over the past 30 years: more than 50% of patients experience recurrence within 2 years, suggesting the importance of enhancing the radiosensitivity of OSCC [4–6].

Hypoxia is the critical cause of radioresistance: oxygen is a source of free radicals, which are needed for ionizing radiation (IR) to kill tumour cells; hypoxia can also induce a series of cellular biological transformations to prevent the harmful effects of IR [7]. When the oxygen tension in the tumour microenvironment (TME) is below 0.13 kPa, radiobiological hypoxia that interferes with radiation-induced cell death occurs [8]. Thus, studies on the mechanism of hypoxia are essential to improve the efficacy of radiotherapy. Existing studies concerning hypoxia and radioresistance have mainly focused on hypoxic cells themselves, rather than on the interactions between hypoxic cells and normoxic cells. Hypoxic regions are typically distributed across the entire viable tumour mass. These hypoxic microregions are arranged on a micron level and exhibit sharp gradients between aerobic and hypoxic areas; because of these two intersecting areas, short-distance intercellular communication is very common [9]. However, the communication between hypoxic cells and normoxic cells during the transfer of radiation resistance remains unclear.

Exosomes and extracellular vesicles (EVs) are small particles of 30–200 nm in diameter that are encapsulated by dual membranes. EVs encapsulate various intercellular signalling molecules, including microRNAs (miRNAs) [10]. Hypoxic tumour cells release specific EVs that can be endocytosed by adjacent cells in the TME, which subsequently causes a series of biological changes [11]. The stable miRNAs capsulated by EVs can function as signalling molecules to confer hypoxia-induced functional alterations such as therapeutic resistance [12]. The combination of hypoxia, EVs and the TME may provide novel insight into radiation resistance. However, data on this relationship are still limited. In the current research, we revealed that hypoxic EVs can facilitate the growth and radioresistance of OSCC cells. We found by high-throughput sequencing that miR-340-5p is highly expressed in hypoxic OSCC EVs. miR-340-5p is directly transferred to normoxic OSCC cells and targets Kruppel-like factor 10 (KLF10), a tumour suppressor. Consequently, miR-340-5p blocks KLF10/UVRAG signalling and IR-induced apoptosis. Knockdown of miR-340-5p in hypoxic EVs can reverse these EV-induced oncogenic effects. Moreover, we demonstrated that the plasma exosomal miR-340-5p level can be used as a biomarker to predict the in-field recurrence of OSCC patients, providing a novel therapeutic target in OSCC.

## Methods

### Patients and specimens

All clinical samples were collected from the Department of Radiation Oncology at the First Affiliated Hospital of Nanjing Medical University. All subjects gave informed consent before they participated in the study. The study was conducted in accordance with the guidelines of the Declaration of Helsinki, and the protocol was approved by the Ethics Committee and Institutional Review Board of the First Affiliated Hospital of Nanjing Medical University. A total of 88 OSCC patients were recruited in this study between 2017 and 2019. Clinical staging was based on the seventh edition of the AJCC Cancer Staging Manual [13]. All patients received a 60 Gy total dose of involved-field radiation (2 Gy per fraction, 5 days per week) plus two cycles of concurrent chemotherapy with cisplatin and fluorouracil and two cycles of post radiation chemotherapy. Radiation was delivered as described previously [14]. Blood samples were collected 1 day prior to definitive chemoradiation. Follow-up studies included computed tomography, barium swallow, endoscopy and physical examination. All patients were followed up every 3 months until death or May 2020. In-field recurrence included primary lesions and involved regional lymph nodes inside the PTV. Overall survival was defined as the interval between completion of radiation and death [14]. We collected 30 ml of whole blood from each patient into BD Vacutainer K2 EDTA Blood Collection Tubes (BD, USA), and we immediately centrifuged the whole blood at 4000×*g* for 10 min at 4 °C to obtain plasma. The plasma was then ultracentrifuged to collect EVs.

### Cell culture and hypoxia treatment

Human OSCC cell lines (Te13, Te1 and Eca109) were obtained from the American Type Culture Collection (ATCC, USA). All cell lines were cultured in RPMI-1640 medium (Gibco, USA) with 10% foetal bovine serum (FBS; Gibco, USA), 100 U/ml penicillin and 100 μg/ml streptomycin. Cells were maintained at 37 °C in 5% CO_2_ and were routinely examined for *Mycoplasma* contamination. To induce hypoxia (< 1% O_2_), cells were cultured at 37 °C in the same incubator in an AnaeroPack jar with AnaeroPack-Anaero (Mitsubishi, Japan) according to the manufacturer’s instructions. The hypoxic environment was confirmed by detection of hypoxia inducible factor 1 subunit alpha (HIF-1α) expression. Cells were irradiated by RS 2000 Pro X-Ray Bio-irradiator (Radsource, USA) with 140 kV X-ray beam. The irradiation field was confined within the culture dish or disk, and the dosing rate was 1.439Gy/min.

### EV isolation and identification

FBS was depleted of EVs by ultracentrifugation at 140,000×*g* and 4 °C for 16 h, and the supernatant was collected and filtered through a 0.22 μm filter (Millipore, USA). EVs derived from blood samples and cell culture medium were isolated by differential centrifugation as previously described [15]. Before EV isolation, cells were cultured in normal medium to 50% confluency and were then washed with phosphate-buffered saline (PBS) three times; the medium was then replaced with RPMI-1640 containing 10% EV-depleted FBS and cultured under normoxic or hypoxic conditions. After 48 h, the cell culture medium was harvested (50 ml), and EVs were isolated by differential centrifugation as previously described. The EVs were used immediately for further experiments. The size distribution and concentration of EVs were analyzed by nanoparticle tracking analysis (NTA) using a ZetaView particle tracker from ParticleMetrix (Meerbusch, Germany). We used transmission electron microscopy (TEM; JEM-1200EX, JEOL Ltd., Japan) to observe the structure of EVs. CD63, CD81 and Alix were used as exosomal markers, and calnexin was used as the negative control marker for EVs. PKH67 (Sigma-Aldrich, USA) was used to label EVs. Twenty-four hours after PKH67-labelled EVs were incubated with OSCC cells, DAPI was used for nuclear staining. Cells were visualized with a confocal fluorescence microscope (Leica, Germany).

### Western blotting

Samples of cells and EVs were washed and resuspended in RIPA lysis buffer (Beyotime, Shanghai, China) with a protease and phosphatase inhibitor mixture (Millipore). Proteins were separated based on their molecular weight by sodium dodecyl sulfate-polyacrylamide gel electrophoresis and then transferred onto polyvinylidene fluoride membranes (Millipore). The membranes were blocked with 5% skim milk powder in Tris-buffered saline containing Tween 20 (TBST) for 2 h, and were then incubated at 4 °C overnight with specific primary antibodies (details are listed in Table S1). The membranes were rinsed in TBST three times (10 min each time), incubated with secondary antibodies at room temperature for 2 h and then washed again in TBST (three times, 10 min each time). Protein expression levels were measured with ECL Plus reagent (Millipore) using a Bio-Imaging System (Bio-Rad, USA).

### Quantitative real-time PCR (qRT-PCR)

Following the manufacturer’s instructions, total RNA was extracted with TRIzol reagent (Invitrogen, USA). For qRT-PCR of mRNA, total RNA was further reverse transcribed into cDNA using a PrimeScript RT Reagent Kit (Takara, Japan). For RT-PCR of miRNA, we used a Mir-X miRNA First-Strand Synthesis Kit (Takara) to perform reverse transcription. The reactions were performed in a real-time PCR system (Applied Biosystems 7500, USA) with a TB Green Premix Ex Taq Kit (Takara). The primers specific for the target mRNAs/miRNAs were obtained from Wcgene Biotech (Shanghai, China). Since hypoxia has an influence on GAPDH expression, β-actin and U6 were used to evaluate mRNA and miRNA levels, respectively, in cell samples [16]. For EV samples, expression levels were normalized to those of the external control cel-miR-39 (RiboBio, Guangzhou, China). The results are shown as relative expression levels calculated by the 2^−ΔΔCT^ method.

### Vector construction, lentivirus production and cell transfections

The LV2-hsa-miR-340-5p-mimic vector and LV2-hsa-miR-340-5p-inhibitor vector were constructed as lentiviral vectors (GenePharma, Shanghai, China). Cells were infected at 40–50% confluence and selected with puromycin to confirm stable transfection. pcDNA3.1 vectors containing KLF10 and the negative control vectors were purchased from GenePharma. siRNAs and mimics were purchased from RiboBio. Lipofectamine 3000 reagent (Invitrogen) was used for transfection. The sequences of the vectors are listed in Table S1.

### Luciferase reporter assay

Sequences corresponding to the 3′-UTR of KLF10 mRNA and containing the wild-type or mutated miR-340-5p binding sequence were synthesized by GeneScript (Nanjing, China). We inserted these sequences into the XbaI and SacI sites in the pmirGLO dual-luciferase miRNA target expression vector (Promega, USA). These vectors were cotransfected with the miR-340-5p-mimic/miR-340-5p-inhibitor or miR-NC by using Lipofectamine 3000 (Invitrogen) according to the manufacturer’s instructions. Luciferase activity was measured with a Luciferase Reporter Assay System (Promega).

### RNA immunoprecipitation (RIP)

An EZ-Magna RIP RNA Binding Protein Immunoprecipitation Kit (Millipore,) was used to conduct the RIP assay. Cells were collected and lysed in pre-cooled lysis buffer supplemented with 1 mM PMSF, protease inhibitor and RNase inhibitor. Cells were then incubated with RIP buffer containing magnetic beads conjugated to a human anti-Argonaute 2 (Ago2) antibody (Millipore) or normal IgG (Millipore). The precipitate was digested, and the co-immunoprecipitated RNA was then isolated for PCR.

### Colony formation assay

We seeded 200, 400, 800, 2000 and 5000 cells/well in 6-well plates 1 day before irradiation with 0, 2, 4, 6 or 8 Gy, respectively. After 2 weeks, each well was washed with PBS for three times, fixed with 4% paraformaldehyde for 30 min and finally stained with crystal violet. Colonies were counted macroscopically. The survival curve was constructed using a single-hit, multi-target model. D0 refers to the mean lethal dose, and it is the dose of radiation that reduces survival to 37%. Dq refers to the quasi-threshold dose, and it is the dose at which the straight portion of the survival curve, extrapolated backward, cuts the dose axis drawn through a survival fraction of unity. SF2 refers to the survival fraction of 2Gy radiation. SER refers to sensitizing enhancement ratio, and is calculated by the ratio of D0 in each condition. GraphPad Prism 8.0 software were used to perform the analysis and the formula required for calculation were listed as follows: SF = 1-(1-*e*^-kd^)^n^; D0 = $\frac{1}{\mathrm{k}}$ ; Dq = D0 × ln n.

### Apoptosis analysis

Apoptosis was assessed using an apoptosis detection kit (Vazyme, Nanjing, China) following the manufacturer’s instructions. At 72 h post irradiation with 8 Gy, cells were digested, resuspended in 500 μl of binding buffer with 5 μl of annexin V-FITC solution and 5 μl of propidium iodide (PI), and stained at room temperature for 15 min in the dark. Flow cytometry (FACScan; BD Biosciences, USA) and FlowJo software (BD, USA) were used to analyze the cells.

### Immunofluorescence assay

A total of 5 × 10^4^ cells were seeded into a confocal laser dish 1 day before irradiation with 8 Gy. Four hours post irradiation, cells were fixed with 4% paraformaldehyde at room temperature for 30 min and permeabilized with 0.1% Triton X-100 for 2 h. Cells were then blocked with 5% BSA for 90 min and washed with PBS. After incubation with the anti-γ-H2AX primary antibody (1:400; Cell Signaling Technology) overnight at 4 °C, cells were washed with PBS and incubated with an Alexa Fluor 555-conjugated secondary antibody (Beyotime) for 90 min. Cells were washed with PBS, treated with DAPI staining solution for 20 min and observed using a confocal fluorescence microscope (Leica).

### Nude mouse xenograft model

This study was approved by the Institutional Animal Care and Use Committee of Nanjing Medical University (IACUC-1901012). BALB/c nude mice were purchased from the Animal Center of Nanjing Medical University. A total of 2 × 10^6^ cells were injected subcutaneously into the flanks of the nude mice. Tumours were measured with calipers every 5 days, and the tumour volume was calculated using the following formula: volume = (width^2^ × length)/2. When the tumour volume reached 50 mm^3^, 10 μg (50 μl) of EVs or 50 μl of PBS was injected into the tumour once daily for 5 days. In addition, tumours were irradiated in an RS 2000 Pro X-Ray Bio-irradiator (Radsource, USA) with a dosage of 2 Gy per day for 4 consecutive days starting on the second day of EV injection. Two weeks post IR, the mice were euthanized. In addition, we injected NC-Te13 cells and miR-340-5p mimic Te13 cells into the flanks of nude mice to study the effect of miR-340-5p. When the tumour volumes reached 50 mm^3^, four fractions of radiation (2 Gy/f) were applied to the tumours, and the mice were sacrificed 14 days after the radiation exposure was completed. Lead shields were used to prevent radiation injury.

### Immunohistochemistry

Immunohistochemistry was performed as previously reported [17]. The staining intensity was scored on a four-grade scale: 0 (no staining), 1 (weak staining), 2 (intermediate staining), or 3 (strong staining). The staining percentages were divided into 4 grades: 0 (no positive cells), 1 (<25% positive cells), 2 (25–50% positive cells) and 3 (>50% positive cells). The product of the staining intensity and staining percentage was used as the final staining score.

### TUNEL assay

The TUNEL assay was performed using an In Situ Cell Death Detection Kit (Roche, Germany). Following the manufacturer’s protocols, sections were deparaffinized and rehydrated. Antigen retrieval was performed using hot 0.1 M citrate buffer (pH 6.0), and incubated with the TUNEL reaction mixture (containing TdT and fluorescein-conjugated dUTP) for 1 h at 37 °C. DAPI was used to stain nuclei. Apoptotic cells were analysed using a fluorescence microscope (Leica). For the negative control reaction, TdT was not included in the reaction mixture.

### Statistical analysis

All experiments in this study were carried out in triplicate. Differences between groups were determined using the non-parametric Kruskal-Wallis test or Mann-Whitney U test with the Bonferroni’s correction. A chi-squared test was used to detect differences in clinical data. The patients were divided into high expression and low expression groups based on the median gene expression level. Kaplan-Meier analysis was used to compare recurrence in OSCC patients. STATA 14.0, SPSS 22.0 and GraphPad Prism 8.0 software were used to perform statistical analysis, and a *p* value < 0.05 was considered statistically significant (*p < 0.05, **p < 0.01, ***p < 0.001).

## Results

### Hypoxic tumour cell-derived EVs alleviate IR-induced apoptosis and promote radioresistance

EVs secreted by three OSCC cell lines were isolated by differential centrifugation. These EVs were clarified by previously described methods [18]: NTA showed that the diameters of all three kinds of EVs were between 60 and 200 nm. TEM was used to observe the structures of EV. The exosomal markers Alix, CD63 and CD81 were detected at high levels in EVs, while calnexin, an endoplasmic reticulum component, was absent in EV samples (Fig. S1A-C). To ensure a hypoxic environment, HIF1-α was used as a marker to confirm hypoxia (Fig. S1D). EVs labelled with PKH67 were endocytosed by recipient cells (Fig. S1E). These data characterize EVs secreted by OSCC cells and indicate that EVs derived from both normoxic and hypoxic OSCC cells can be endocytosed.

**Fig. 1:**
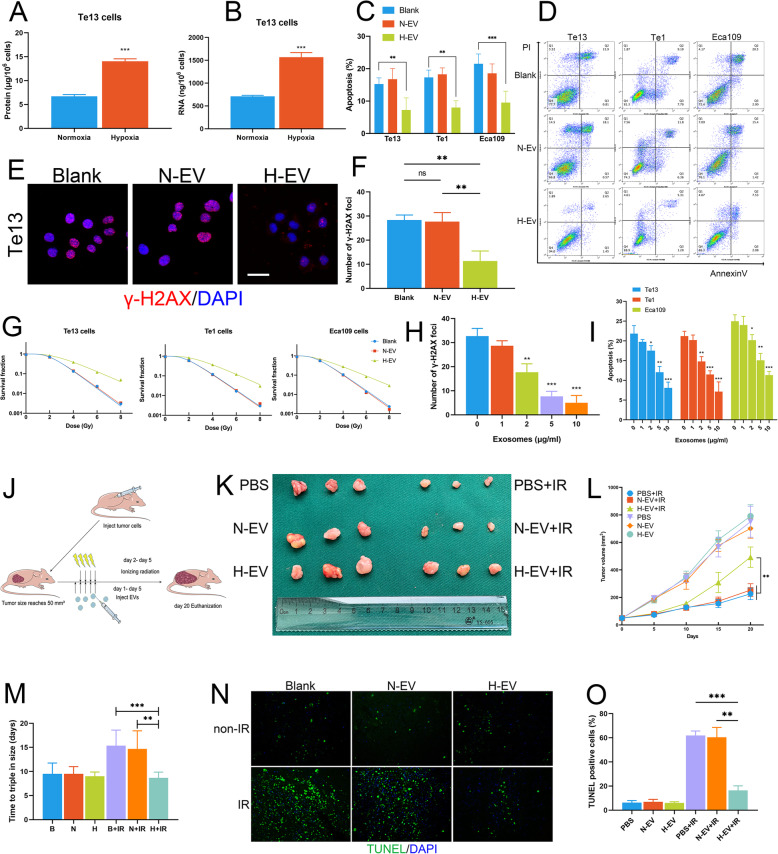
EVs derived from hypoxic OSCC cells promote radioresistance. **a-b** EVs derived from OSCC cells in a hypoxic environment contain higher levels of protein (**a**) and RNA (**b**). **c-d** Coculture with H-EVs drastically decreased OSCC cells apoptosis induced by irradiation. **e-f** H-EV-treated OSCC cells expressed lower levels of the DNA damage repair marker γ-H2AX after irradiation (scale bar = 20 μm). **g** Cell survival curve constructed from the colony formation assay data. Cells were supplemented with N-EVs or H-EVs and ionizing radiation as indicated. **h** The expression of γ-H2AX in irradiated OSCC cells was related to H-EV supplementation in a dose-dependent manner (scale bar = 20 μm). **i** Apoptosis of OSCC cells caused by irradiation was decreased by coculture with H-EVs in a dose-dependent manner. **j** Schematic diagram of the experimental design used to establish the animal model. **k** Images of tumours in each group (*n* = 6). **l** Alterations in the tumour volume in each group (*n* = 6). **m** Time to triple the tumour volume after the first day of EV injection in each group (*n* = 6). **n-o** H-EV-treated OSCC xenografts exhibited fewer TUNEL positive cells after irradiation

Next, we found that EVs derived from OSCC cells under hypoxic conditions contained higher levels of protein and RNA than those isolated under normoxic conditions, indicating that hypoxia may cause alterations in the EV content (Fig. 1a-b). To determine the functions of EVs derived under the two different conditions, we cocultured OSCC cells with N-EVs and H-EVs. It was shown that H-EVs, but not N-EVs, drastically decreased apoptosis in treated cells after IR (Fig. 1c-d). In addition, the expression of the DNA damage repair protein γ-H2AX was significantly decreased in H-EV-treated cells after IR (Fig. 1e-f). The cell survival curve constructed from the colony formation assay results (Fig. 1g) revealed the radioresistant effect of H-EV (Fig. S2G). To study the direct role of EVs in OSCC cells, we used GW4869, a widely used exosome secretion inhibitor, to reduce EV production. We found that GW4869 inhibited colony formation by OSCC cells after IR but had no effect on non-irradiated cells. In addition, GW4869 decreased colony formation and increased apoptosis only after IR of cells treated with H-EVs (Fig. S2A-B). The radiosensitivity values assessed from cell survival curve showed the same results (Fig. S2C-D). Additionally, the DNA damage caused by IR was alleviated in H-EV-cocultured cells, as detected by γ-H2AX expression, as the concentration of H-EV increased. (Fig. 1h, Fig. S2F). Similarly, the apoptosis induced by IR and the colony formation ability after IR showed the same results in H-EV- treated cells in a dose-dependent manner (Fig. 1i, Fig. S2E). These results indicate that EVs secreted by hypoxic OSCC cells can promote radioresistance in normoxic cells.

We further verified the role of H-EVs in radioresistance in a xenograft mouse model (Fig. 1j). We found that injection of H-EVs but not N-EVs or PBS drastically enhanced tumour growth only in the IR group and not in the non-irradiated group (Fig. 1k-l). The time required for xenografts to triple in size after the last day of IR was significantly decreased in the irradiated H-EV group (Fig. 1m). Moreover, IR-induced cell damage and apoptosis occurred, as evidenced by the TUNEL-positive cells, and were significantly reduced by H-EV supplementation (Fig. 1n-o). These results confirm that hypoxic tumour cell-derived EVs can inhibit IR-induced apoptosis and promote radioresistance.

### miR-340-5p is highly expressed in H-EVs and promotes radioresistance in OSCC

**Fig. 2:**
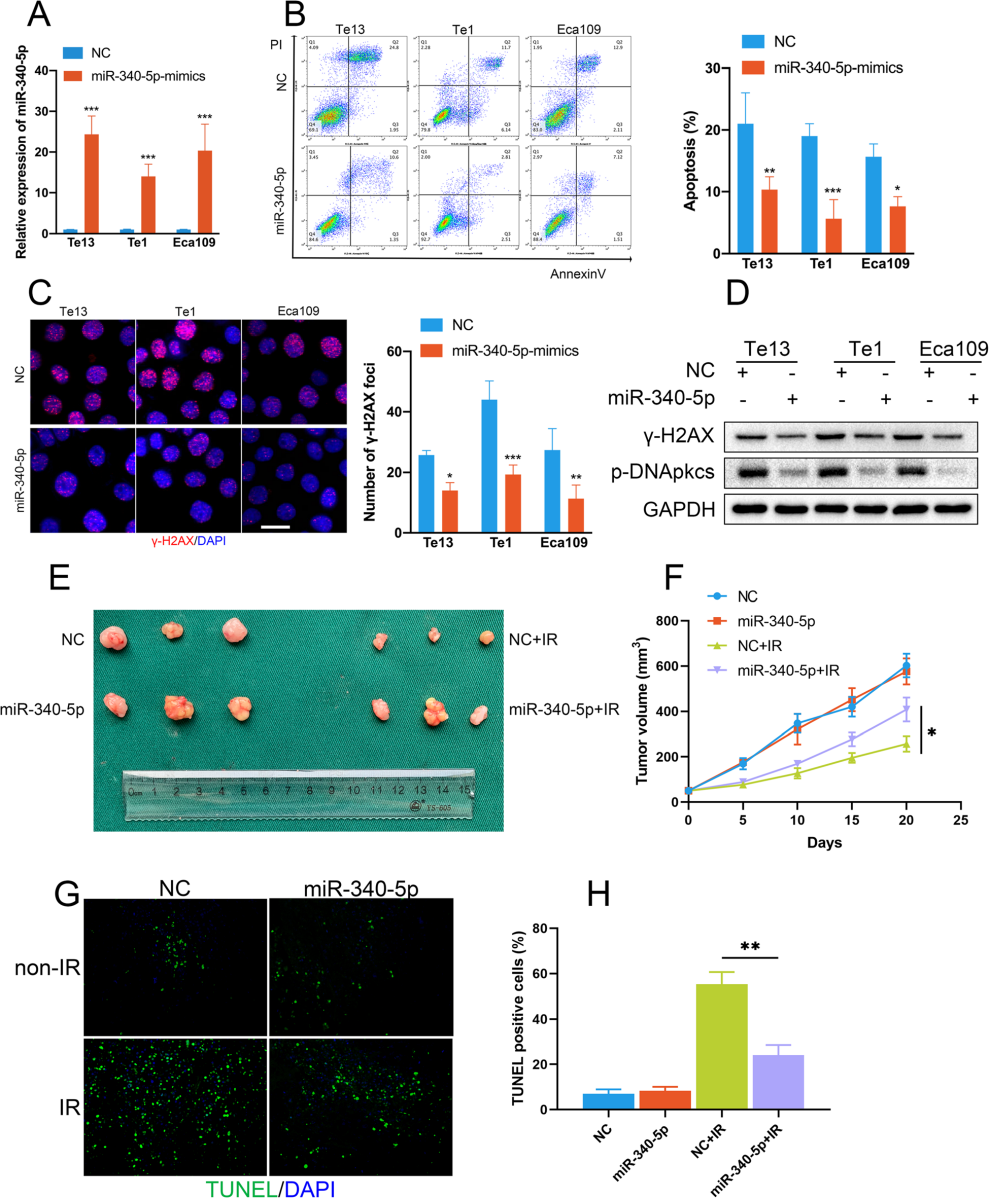
miR-340-5p promotes radioresistance in OSCC. **a** qRT-PCR confirmed the effect of miR-340-5p mimic transfection. **b** miR-340-5p overexpression suppressed irradiation-induced apoptosis in OSCC cells. **c** The expression of γ-H2AX was decreased in miR-340-5p overexpressing OSCC cells after irradiation (scale bar = 20 μm). **d** Western blot assays demonstrated that miR-340-5p blocked the expression of the DNA damage repair proteins γ-H2AX and p-DNApkcs after irradiation in OSCC cells. **e** Images of tumours in each group (*n* = 6). **f** Alterations in the tumour volume in each group (*n* = 6). **g-h** miR-340-5p-overexpressing OSCC xenografts exhibited fewer TUNEL positive cells after irradiation. NC: cells transfected with negative control lentiviral vectors; miR-340-5p: cells transfected with miR-340-5p mimic lentiviral vectors

Because miRNAs are important intercellular signalling molecules encapsulated by EVs, we inferred that they may be associated with H-EV-induced radioresistance in OSCC. We previously reported high-throughput miRNA-seq results in EVs derived from both normoxic and hypoxic conditions of OSCC cells, finding that miR-340-5p is highly expressed in H-EVs derived from OSCC cells [15]. Previous studies have reported that miR-340-5p plays a dual role in cancer development and progression. Additionally, there are studies showing that miR-340-5p is related to the invasion and migration abilities of OSCC cells. To further investigate the role of miR-340-5p in radiosensitivity, we transfected miR-340-5p mimic lentiviral vectors into OSCC cells. The transfection efficiency was verified by qRT-PCR (Fig. 2a). Surprisingly, miR-340-5p mimics transfection drastically decreased apoptosis after IR (Fig. 2b). In addition, we found that miR-340-5p greatly reduced DNA damage after IR (Fig. 2c-d). Previous studies have indicated that miR-340-5p acts as both an inhibitor of migration and invasion and a pro-cancer factor in promoting proliferation [19, 20]. While those results were derived only from in vitro experiments, we established tumour xenograft mouse models to study the in vivo functions of miR-340-5p in OSCC. After transfection with miR-340-5p mimics lentiviral vectors, the cells were subcutaneously injected into mice as described above. Interestingly, we determined that overexpression of miR-340-5p did not change proliferation in the non-IR groups but significantly promoted tumour growth in the IR groups (Fig. 2e-f). In addition, miR-340-5p reduced apoptosis after IR, as determined by TUNEL assays (Fig. 2g-h). These results demonstrate that miR-340-5p decreases IR-induced apoptosis and DNA damage and promotes radioresistance in OSCC.

### miR-340-5p is essential for the hypoxic EV-induced radioresistance shift in OSCC

**Fig. 3:**
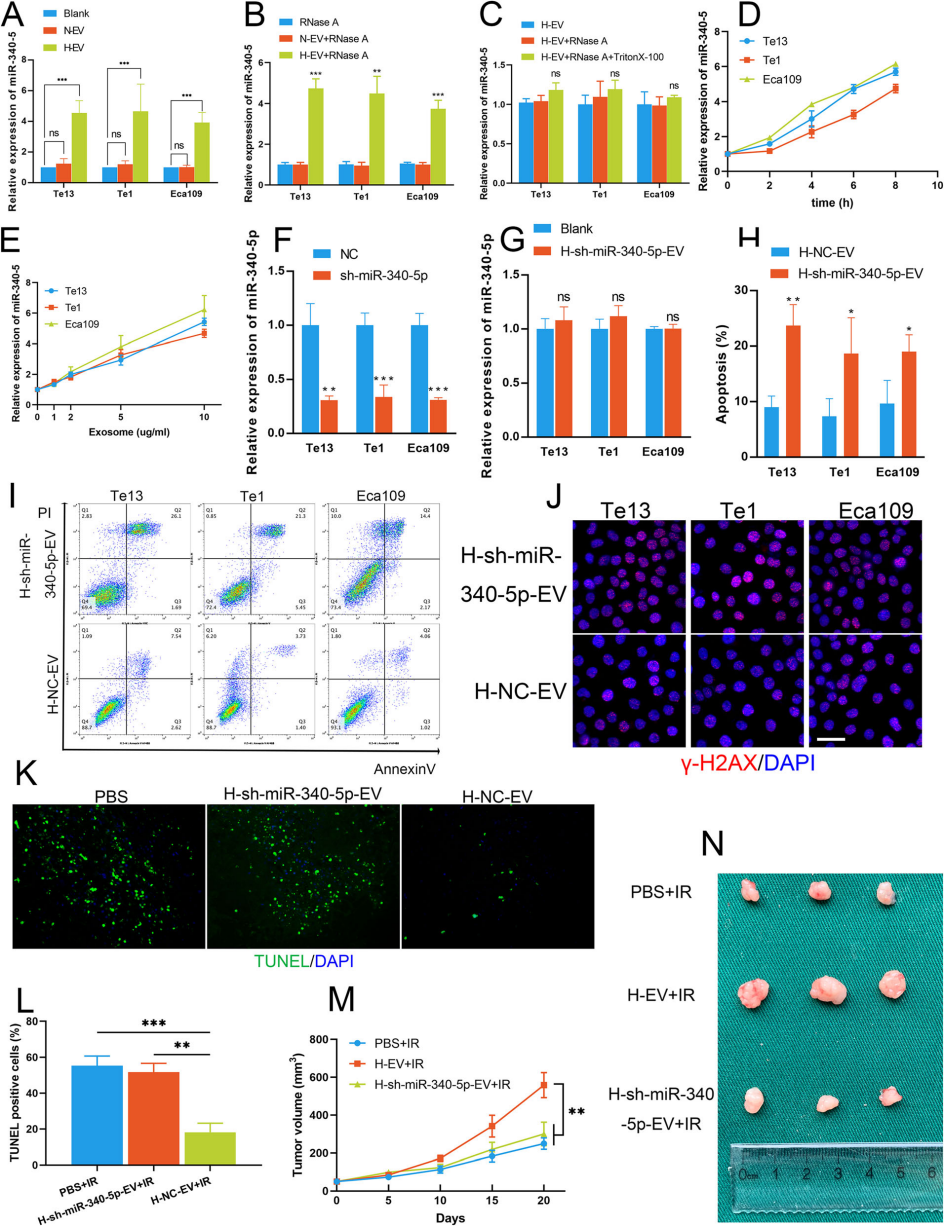
miR-340-5p is essential for the hypoxic EV-induced radioresistance shift in OSCC. **a** The miR-340-5p level was greatly elevated in H-EV-treated OSCC cells but not in N-EV-treated OSCC cells. **b** qRT-PCR revealed miR-340-5p expression in OSCC cells after coculture with RNase A-treated EVs. **c** miR-340-5p levels in OSCC cells treated with RNase A or with RNase A and Triton X-100 together, as detected by qRT-PCR assays. **d-e** The expression of miR-340-5p in OSCC cells was associated with H-EV supplementation in a time-dependent (**d**) and dose-dependent (**e**) manner. **f** qRT-PCR confirmed the effect of miR-340-5p knockdown in OSCC cells. **g** No significant changes were observed by qRT-PCR between OSCC cells cocultured with PBS or H-sh-miR-340-5p-EVs. **h-i** Knockdown of miR-340-5p in hypoxic EVs rescued the suppression of IR-induced apoptosis caused by H-EVs. **j** Knockdown of miR-340-5p in hypoxic EVs reversed the H-EV-induced decrease in γ-H2AX expression after irradiation in OSCC cells (scale bar = 20 μm). **k-l** Knockdown of miR-340-5p in hypoxic EVs reversed the decreased in TUNEL-positive cell numbers in OSCC xenografts after irradiation. **m** Alterations in the tumour volume in each group (*n* = 6). **n** Images of tumours in each group (*n* = 6). NC: cells transfected with negative control lentiviral vectors

Having determined the direct function of miR-340-5p in OSCC radiosensitivity, we inferred that miR-340-5p is critical for H-EV-induced radioresistance transfer in OSCC. To study whether miR-340-5p is transferred as EV cargo, we cocultured OSCC cells with PBS, N-EVs and H-EVs. We found that the levels of miR-340-5p was greatly increased in H-EV-treated cells but not in N-EV-treated cells compared to that in PBS-treated cells (Fig. 3a). To rule out possible effections of free RNAs in the supernatants, we used RNase A to remove residual RNA contaminants in the supernatant of EV extracts and found that the PCR results did not change (Fig. 3b). Moreover, we used Triton X-100 and RNase A together to destroy RNA encapsulated within the EV membrane, and we found that this treatment reversed the increase of the miR-340-5p level in H-EV-treated cells (Fig. 3c). The increase of miR-340-5p level in H-EV recipient cells occurred in a time-dependent and dose-dependent manner (Fig. 3d-e). These results indicate that miR-340-5p is encapsulated in EVs and can be delivered from H-EVs to the recipient OSCC cells.

To further investigate the function of miR-340-5p in H-EV-induced radioresistance, we knocked down miR-340-5p in OSCC cells (Fig. 3f). EVs secreted under hypoxic conditions by miR-340-5p knockdown cells (H-sh-miR-340-5p-EVs) and NC cells (H-NC-EVs) were extracted. The q-PCR results showed that miR-340-5p expression did not change in cells treated with H-sh-miR-340-5p-EVs compared with that in cells treated with H-NC-EVs (Fig. 3g). Next, we cocultured OSCC cells with H-NC-EVs and H-sh-miR-340-5p-EV. We found that uptake of H-sh-miR-340-5p-EVs rescued the suppression of IR-induced apoptosis caused by H-EV treatment (Fig. 3h- i). Additionally, it was shown that H-sh-miR-340-5p-EVs lost the ability to reduce the expression of γ-H2AX in recipient cells (Fig. 3j). In vivo studies showed the same results: H-sh-miR-340-5p-EV treatment reversed the decreased in TUNEL-positive cells in xenografts after IR (Fig. 3k-l). In addition, injection of H-sh-miR-340-5p-EVs into nude mice did not produce a radioresistance effect similar to that seen in the H-EV-injected group (Fig. 3m-n). Taken together, these results confirm that miR-340-5p is essential for the hypoxic EV-induced radioresistance shift in OSCC.

### miR-340-5p induces radioresistance by affecting the KLF10/UVRAG axis

**Fig. 4:**
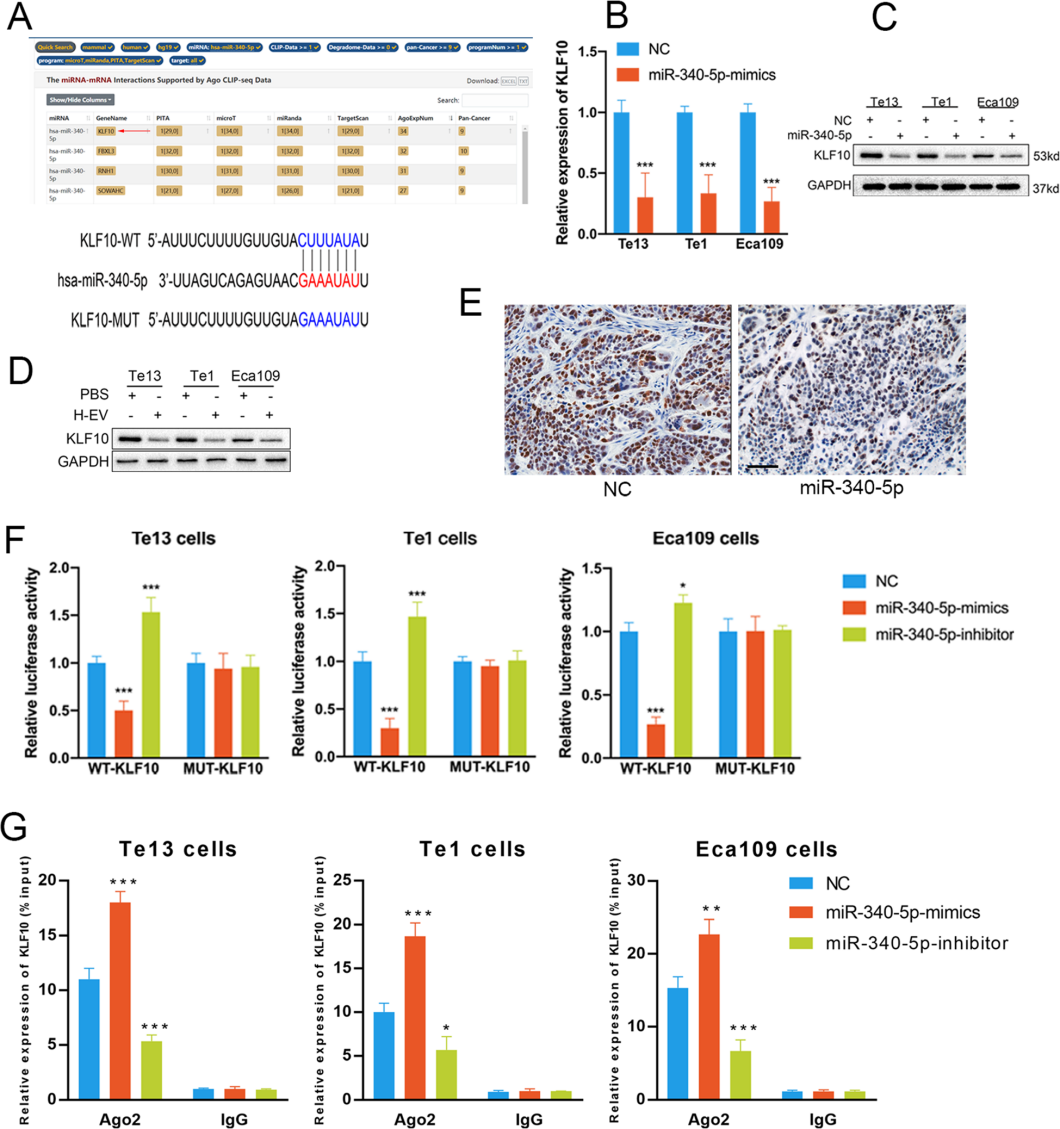
KLF10 is a direct target for miR-340-5p. **a** Illustration of the putative predicted miR-340-5p binding site in the KLF10 mRNA 3′-UTR. **b-c** Expression of KLF10 in OSCC cells transfected with relevant lentiviral vectors was detected by qRT-PCR (**b**) and Western blotting (**c**). **d** Expression of KLF10 in OSCC cells was decreased after cocultured with hypoxic EVs. **e** Expression of KLF10 was detected by IHC in xenografts transfected with the relevant lentiviral vectors (scale bar = 200 μm). **f** Luciferase reporter assays were conducted to verify that miR-340-5p binds to the 3′-UTR region of KLF10 directly. **g** RIP assays confirmed the binding status between miR-340-5p and KLF10 in treated and untreated OSCC cells. NC: cells transfected with negative control lentiviral vectors; miR-340-5p: cells transfected with miR-340-5p mimic lentiviral vectors

We employed a bioinformatic analysis approach with starBase (http://starbase.sysu.edu.cn/) [21] to investigate the downstream targets of miR-340-5p. The criteria for selection was prediction in at least 9 cancer types and identification by all 4 prediction programs (microT, miRanda, PITA and TargetScan). We found that KLF10 ranked first in the prediction list, and a presumed binding site for miR-340-5p was identified in the KLF10 3′-UTR (Fig. 4a). qRT-PCR and Western blot analyses revealed decreased KLF10 levels in miR-340-5p-overexpressing OSCC cells (Fig. 4b-c). In addition, we found that H-EV-treated cells exhibited lower KLF10 levels than PBS-treated cells (Fig. 4d). The IHC results implied that overexpression of miR-340-5p reduced the number of KLF10-positive cells in xenografted mice (Fig. 4e). We next carried out dual luciferase reporter assays to validate our assumption. Luciferase activity was considerably decreased in cells expressing the miR-340-5p mimic and wild-type KLF10. In contrast, luciferase activity was elevated in cells expressing the miR-340-5p inhibitor and wild-type KLF10. However, luciferase activity was not drastically altered in cells expressing the miR-340-5p mimic/inhibitor and mutant KLF10 (Fig. 4f). In cells expressing the miR-340-5p mimic, KLF10 was upregulated, while in cells expressing the miR-340-5p inhibitor, KLF10 was downregulated, as revealed by the RIP results (Fig. 4g). These results suggest that KLF10 is a direct target of miR-340-5p.

**Fig. 5:**
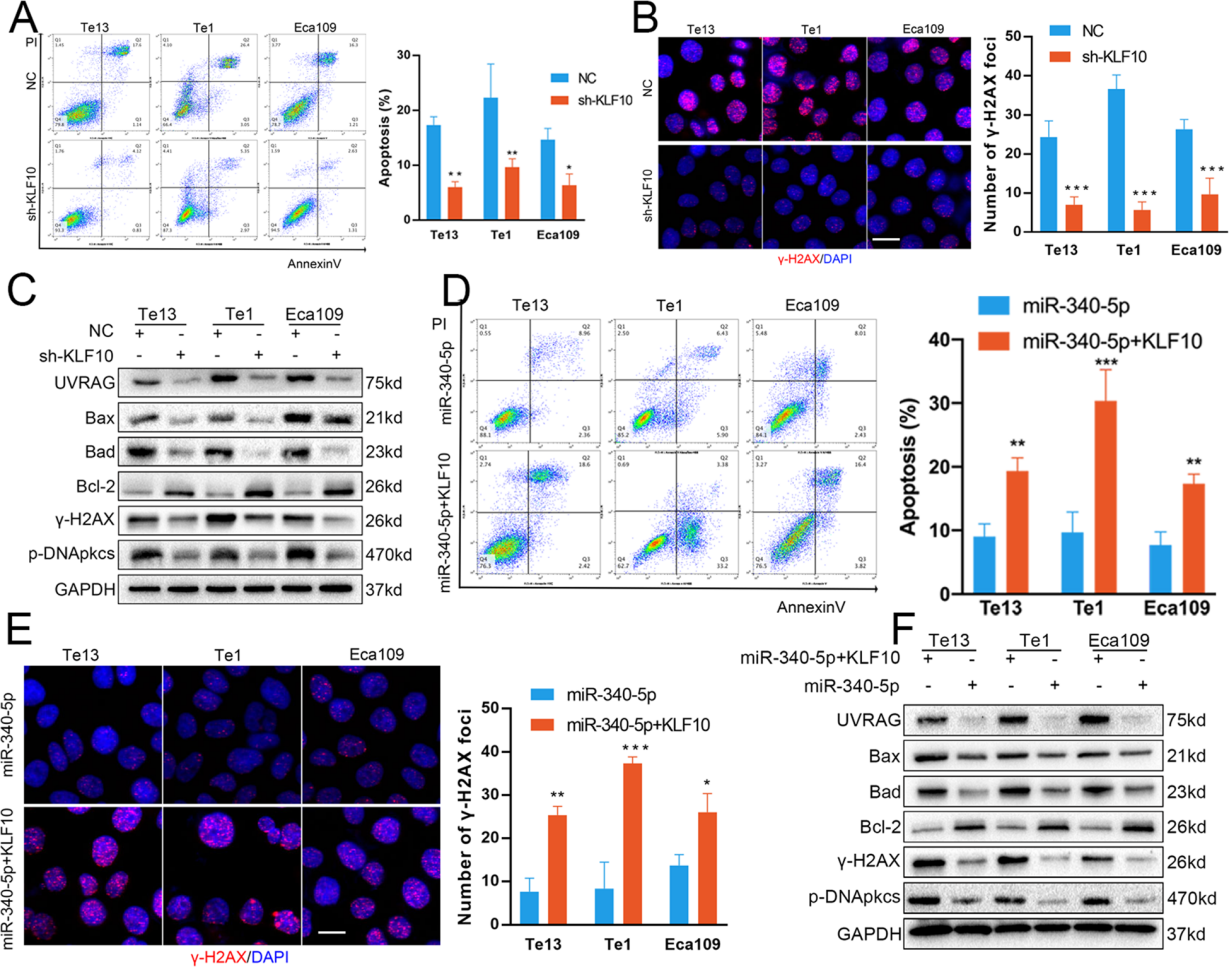
miR-340-5p induces radioresistance by affecting KLF10. **a** KLF10 knockdown attenuated apoptosis caused by irradiation in OSCC cells. **b** KLF10 knockdown decreased the expression of the DNA damage repair protein γ-H2AX (scale bar = 20 μm). **c** Western blot analysis was used to detect the expression of several key factors involved in apoptosis and DNA damage repair in OSCC cells transfected with KLF10 knockdown and control lentiviral vectors. **d-f** Overexpression of KLF10 in OSCC cells transfected with the miR-340-5p mimic lentiviral vectors rescued the apoptosis (**d**), DNA damage repair (**e**, scale bar = 20 μm), and the expression of several key proteins after irradiation (**f**). NC: cells transfected with negative control lentiviral vectors; sh-KLF10: cells transfected with KLF10 knockdown lentiviral vectors; miR-340-5p: cells transfected with miR-340-5p mimic lentiviral vectors; miR-340-5p + KLF10: cells cotransfected with miR-340-5p mimic and KLF10 mimic lentiviral vectors

After identifying KLF10 as a genuine downstream target of miR-340-5p, we investigated the biological function of KLF10 in the radioresistance of OSCC cells. First, we generated a KLF10 inhibitor lentiviral vector and transfected it into OSCC cells. The results of cell-based experiments indicated that silencing KLF10 greatly reduced the apoptosis of OSCC cells after IR (Fig. 5a). Knockdown of KLF10 protein expression also accelerated DNA damage repair after IR (Fig. 5b). UV radiation resistance-associated gene (UVRAG) has been widely recognized as an important player in autophagy. However, it was recently reported to be associated with chromosomal stability independent of its role in autophagy. Previous studies have reported that KLF10 can transcriptionally suppress UVRAG promoter activity, which subsequently affects radiosensitivity by modulating some key factors involved in apoptosis and DNA repair [22]. In our study, miR-340-5p directly targeted KLF10, and we sought to determine whether miR-340-5p can modulate any key proteins in apoptosis and DNA damage repair in OSCC cells. We detected some key factors in apoptosis and DNA damage repair by immunoblotting. As shown in Fig. 5c, silencing KLF10 decreased the expression of UVRAG, Bax and Bad but increased the expression pf Bcl-2 in OSCC cells. Additionally, silencing KLF10 repressed γ-H2AX and p-DNApkcs expression. Collectively, these findings show that KLF10 is a tumour suppressor and is crucial for IR-induced apoptosis and DNA damage repair. To further elucidate the role of KLF10 in miR-340-5p-mediated radioresistance, we co-transfected miR-340-5p mimic and KLF10 mimic lentiviral vector into OSCC cells. Rescue experiments confirmed that the radioresistance promoted by miR-340-5p overexpression was counteracted by ectopic KLF10 expression (Fig. 5d-e). In addition, ectopic KLF10 expression rescued the miR-340-5p-induced changes in the levels of key proteins in apoptosis and DNA damage repair (Fig. 5f). Thus, these results indicate that miR-340-5p promotes growth and radioresistance by directly targeting the KLF10/UVRAG axis.

### Metformin increased KLF10 expression and enhanced the radiosensitivity of OSCC

**Fig. 6:**
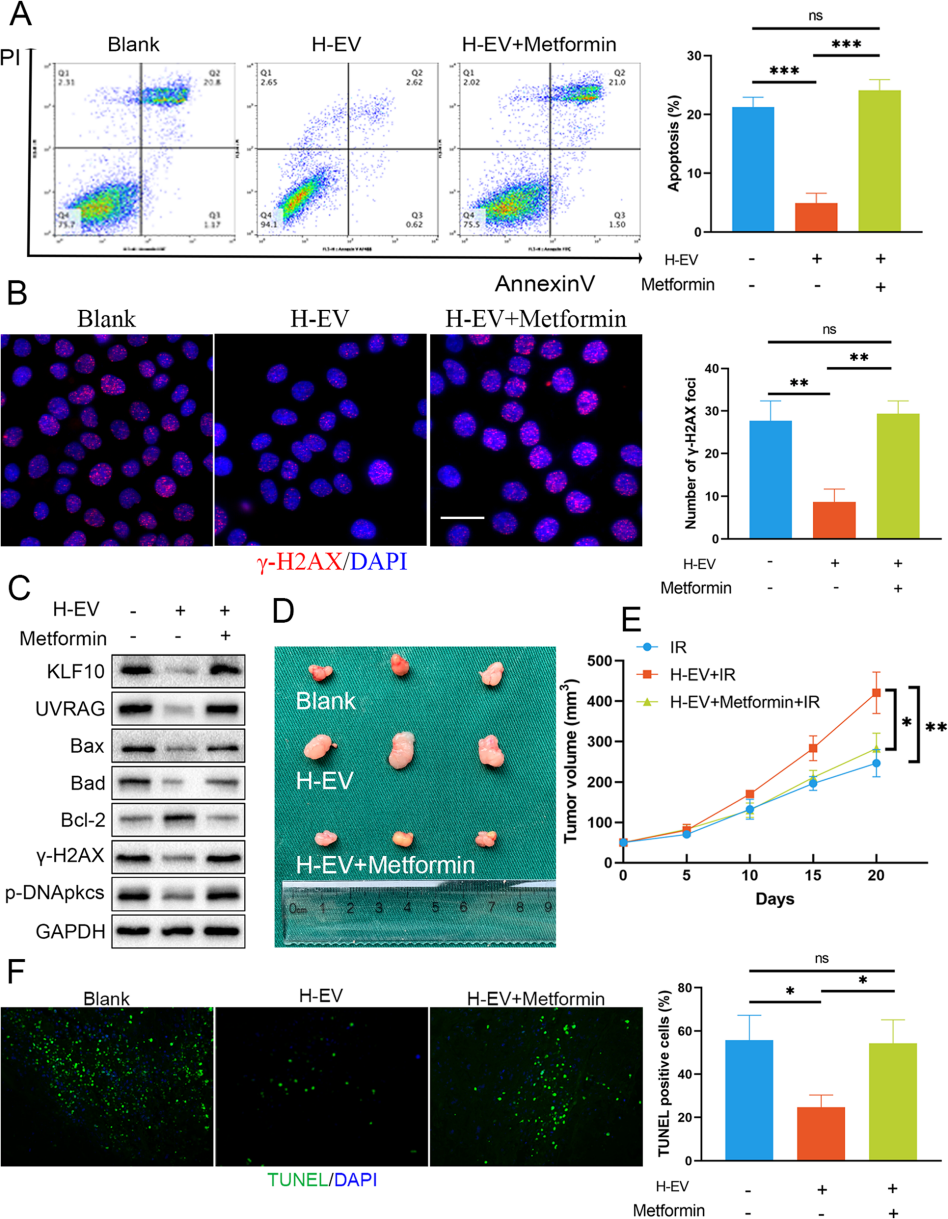
Metformin increased KLF10 expression and enhanced the radiosensitivity of OSCC cells. **a-b** Metformin reversed the decreased apoptosis (**a**) and accelerated DNA damage repair (**b**, scale bar = 20 μm) after irradiation caused by coculture with H-EVs. **c** Metformin increased KLF10 expression and regulated several key factors in irradiated OSCC cells treated with H-EVs. **d** Images of tumours in each group (*n* = 6). **e** Alterations in the tumour volume in each group (*n* = 6). **f** TUNEL-positive cell numbers were decreased after metformin supplementation

Previous studies indicated that the incidence of metabolic diseases is increased in KLF10 knockout mice and that AMPK can phosphorylate KLF10 [22]. Metformin-induced activation of the energy sensor AMPK is well documented. Since KLF10 depletion accounted for the radioresistance induced by H-EVs, we tried to use metformin to upregulate KLF10. We first tested the effect of metformin treatment on IR-induced apoptosis and DNA damage repair in vitro. We found that metformin drastically reversed the decreased apoptosis and accelerated DNA damage repair after IR caused by coculture with H-EVs (Fig. 6a-b). Next, we used immunoblot analysis to confirm whether metformin increases KLF10 expression and regulates several key factors in irradiated OSCC cells treated with H-EVs (Fig. 6c). Injection of H-EV and metformin together in nude mice reversed the radioresistance effect observed in the H-EV group (Fig. 6d-e). In addition, in vivo studies showed the same results: metformin reversed the decrease in TUNEL-positive cell numbers in xenografts after IR in the H-EV group (Fig. 6f). These results indicate that metformin reverses the decrease in KLF10 expression caused by H-EV supplementation and can enhance radiosensitivity in OSCC.

### Upregulation of plasma exosomal miR-340-5p indicates radioresistance and correlates with a poor response to radiotherapy in OSCC patients

**Fig. 7:**
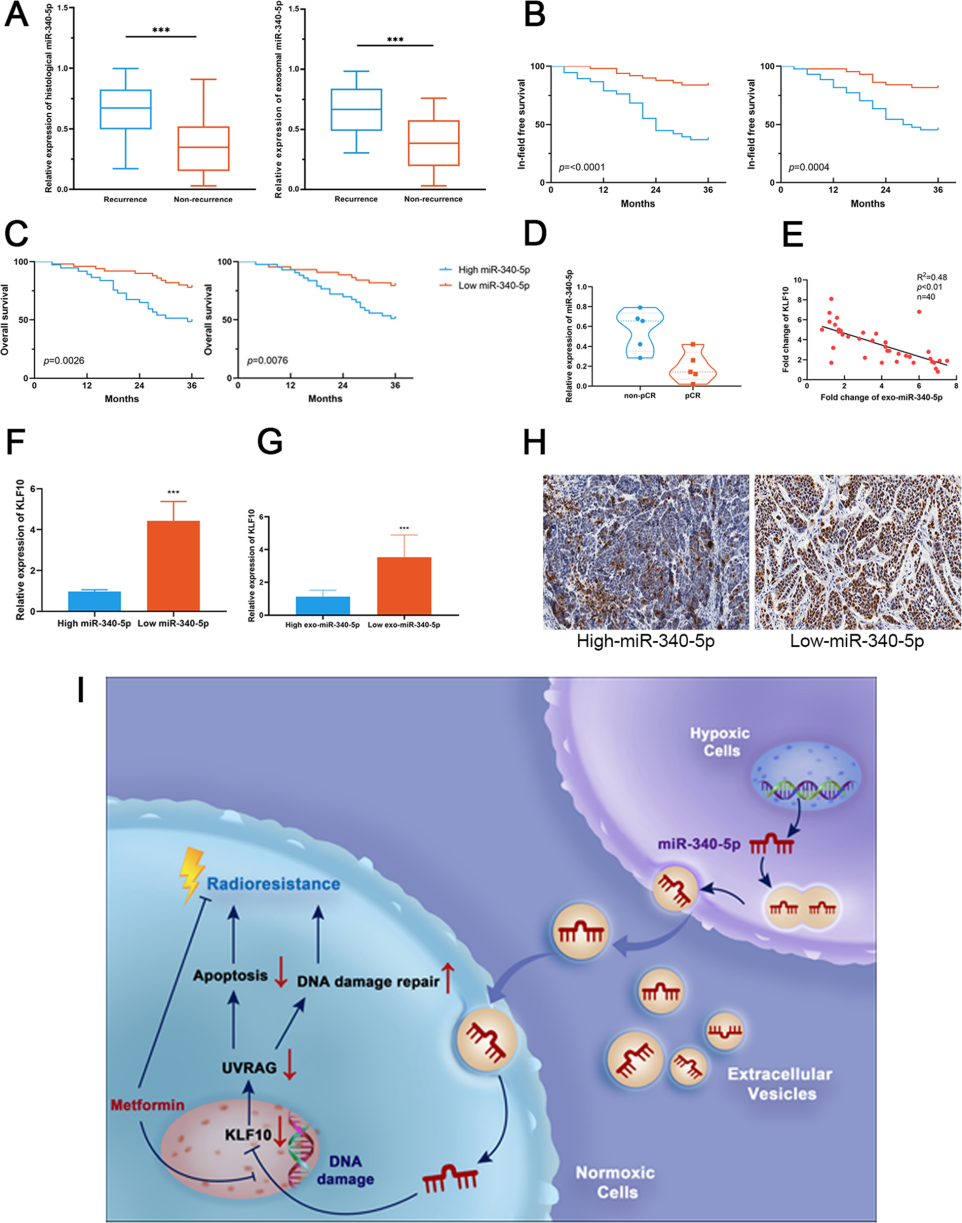
Upregulation of miR-340-5p indicates radioresistance and correlates with a poor response to radiotherapy in OSCC patients. **a** qRT-PCR analysis of miR-340-5p levels in tissues and plasma exosomes of OSCC patients with and without in-field recurrence within 3 years post radiotherapy. **b-c** Kaplan-Meier survival analysis of in-field recurrence free survival (**b**) and overall survival (**c**) of OSCC patients with different histological and plasma exosomal miR-340-5p levels 3 years post radiotherapy. **d** Expression of plasma exosomal miR-340-5p in 10 OSCC patients receiving neoadjuvant chemoradiotherapy. **e** Expression of KLF10 was negatively correlated with the plasma exosomal miR-340-5p levels in 40 OSCC patients. **f-g** Expression of KLF10 in OSCC tissues (**f**) and plasma EVs (**g**) was detected by qRT-PCR in clinical samples of patients with different miR-340-5p levels. **h** IHC showing the expression of KLF10 in clinical specimens expressing different miR-340-5p levels. **i** Schematic diagram showing that metformin attenuates hypoxic tumour cell-orchestrated radioresistance cascades

Having determined that EVs can induce radioresistance in OSCC cells by transferring miR-340-5p, we sought to evaluate the predictive value of miR-340-5p in the radiotherapy response and survival after radiation in OSCC patients. We analysed histological miR-340-5p levels and plasma exosomal miR-340-5p levels in 88 OSCC patients receiving definitive chemoradiotherapy. Details of the patients’ clinical characteristics are listed in Table S2. We identified that OSCC patients who had in-field recurrence within 3 years post radiotherapy exhibited higher expression levels of both histological and plasma exosomal miR-340-5p (Fig. 7a). Higher levels of both histological and plasma exosomal miR-340-5p were also associated with poorer in-field recurrence-free survival and overall survival of OSCC patients, suggesting that miR-340-5p plays a pro-cancer role in OSCC (Fig. 7b-c). In addition, in a preliminary study, we found that the expression of plasma exosomal miR-340-5p in OSCC patients can be used to predict the pathological complete remission (pCR) rate after receiving neoadjuvant chemoradiotherapy (Fig. 7d). However, the number of enrolled patients was quite small, and more studies concerning this issue are needed in the future. Moreover, q-PCR analysis confirmed that the expression of KLF10 in OSCC tissue is negatively related to the levels of both histological and plasma exosomal miR-340-5p in OSCC patients (Fig. 7e-g). IHC staining of OSCC biopsies further showed that the protein expression of KLF10 was greatly increased in patients with low histological miR-340-5p expression levels (Fig. 7h). Considering these results collectively, we conclude that miR-340-5p is a pro-cancer factor in OSCC and can be used as a biomarker to predict the prognosis of OSCC patients.

## Discussion

Radiotherapy has been widely accepted as an essential treatment for cancers and is the first-line strategy for patients with locally advanced OSCC [23, 24]. Unfortunately, radiation resistance is becoming increasingly severe and remains one of the major limitations in OSCC clinical therapy [25, 26]. Therefore, investigating the molecular mechanisms underlying radioresistance may be of great significance for improving OSCC patient outcomes. Hypoxia is a critical cause of radioresistance [27]. Although the radioresistance mechanisms of hypoxic cells have been extensively studied, the complexity of the hypoxic response in the hypoxic TME needs to be defined more clearly.

EVs, including exosomes, which are small membrane vesicles 30–200 nm in diameter, contain numerous signalling molecules. Tumour-derived EVs may mediate cellular communication, stimulating tumour proliferation and therapeutic resistance [28]. In the hypoxic TME, depletion of oxygen and the acidic milieu can activate HIF-1α, which subsequently causes downstream protein expression changes in hypoxic cells. These changes lead to the production of large amounts of exosomes as well as alterations in exosomal nucleic acid profiles. EVs are endocytosed by normoxic cells, and the encapsulated nucleic acids are also transferred to normoxic cells. Consequently, a series of biological changes occur in normoxic cells [12]. Many studies have demonstrated that exosomal miRNAs play important roles in cancer biology, with both diagnostic and therapeutic implications. miRNAs can be horizontally transferred into normoxic cells through hypoxic EVs to modify the TME. A bystander effect caused by these transmitted hypoxic exosomal miRNAs can promote the malignant transformations of normoxic cells [29].

In this study, we extracted EVs from OSCC cells in oxygen-depleted conditions by differential ultracentrifugation. In vivo and in vitro studies revealed accelerated proliferation and radioresistance of normoxic OSCC cells after incubation with hypoxic EVs, suggesting a cancer-promoting effect. In addition, we previously employed high-throughput sequencing techniques to identify the differentially expressed miRNAs in the H-EVs derived from Te13 cells, and we previously found that the miR-340-5p level was elevated in all three tested types of OSCC cell lines [15]. miR-340-5p is known as both a tumour inhibitor and a tumour promoter in multiple cancers, including breast cancer [30], thyroid cancer [31], gastric cancer [32] and non-small cell lung cancer [33]. Previous studies have indicated that miR-340-5p acts as both an inhibitor of migration and invasion and a pro-cancer factor in promoting cell proliferation in OSCC [19, 20]. While those results were derived only from in vitro results, we established a xenograft mouse model to study the in vivo functions of miR-340-5p in OSCC, and we found that miR-340-5p promotes radioresistance. Moreover, miR-340-5p is critical for the H-EV-induced transfer of radioresistance in OSCC. To further investigate the mechanisms of miR-340-5p in OSCC, we employed bioinformatic analysis to study its downstream targets. Among the potential mRNA targets, KLF10 ranked first. The KLF family has been discovered to be involved in essential cellular activities, including proliferation, differentiation, programmed cell death, and neovascularization [34]; therefore, this family is involved in numerous aspects of tumorigenesis. In our research, we proved that KLF10 is a genuine target of miR-340-5p. The increased miR-340-5p in OSCC degraded KLF10 mRNA, while KLF10 reversed the effects of proliferation initiation and apoptosis inhibition caused by exosomal miR-340-5p both in vivo and in vitro.

KLF10 has been reported to transcriptionally suppress UVRAG promoter activity, which subsequently affects radiosensitivity by modulating some key factors involved in apoptosis and DNA repair. A binding site located in the UVRAG promoter region was confirmed to be inhibited by KLF10 binding [22]. In our research, we demonstrated that upregulation of miR-340-5p and knockdown of KLF10 can decrease the expression of UVRAG, Bax and Bad but increase that of Bcl-2 in OSCC cells. Additionally, silencing KLF10 repressed γ-H2AX and p-DNApkcs expression. Metformin, a well-known drug for diabetes, has been reported to improve the response to therapy and reprogram the tumour immune microenvironment in OSCC [35]. Metformin can induce KLF10 expression via AMPK and increase the radiosensitivity of pancreatic cancer cells. In our study, we found that metformin can enhance radiosensitivity by inducing KLF10 expression in H-EV-treated OSCC cells. Additionally, in vivo results confirmed its radiosensitizing effect, indicating its potential application in OSCC radiotherapy.

Having confirmed that hypoxic EVs and miR-340-5p are pro-cancer factors, we aimed to investigate their clinical value. We found that plasma exosomal miR-340-5p expression is positively associated with in-field recurrence in OSCC. In addition, miR-340-5p levels in plasma EVs showed encouraging accuracy in predicting radioresistance and prognosis. Liquid biopsy is currently used to monitor treatment response and predict prognosis in patients with solid tumours. EVs containing miRNAs appear stable in plasma and are promising biomarkers for cancers [36]. miRNAs contained in exosomes secreted by hypoxic tumour cells can reflect hypoxia in the entire TME. Compared with traditional imaging with hypoxia probes, plasma exosomes more accurately reflect the effects of the hypoxic microenvironment on cell biological functions, which may be a more important reason for hypoxia-induced radioresistance.

## Conclusions

Our studies demonstrate that exosomes from hypoxic OSCC cells confer radioresistance by transferring miR-340-5p. miR-340-5p can block IR-induced apoptosis by negatively regulating KLF10 and UVRAG. Metformin can block radioresistance transfer by upregulating KLF10. Clinical specimens of OSCC demonstrated the value of plasma exosomal miR-340-5p in predicting the radiotherapy response, providing novel insight into whether this miRNA is a potential biomarker and a therapeutic target in OSCC patients.

## Supplementary Information

**Additional file 1: Table S1. **Antibody information and vector sequences**.**

**Additional file 2: Table S2.** Clinical characteristics of OSCC patients.

**Additional file 3: Figure S1.** Characterization of OSCC EVs. **A** Nanoparticle tracking analysis showing that the diameters of EVs extracted from OSCC cells were mainly between 60 and 180 nm. **B** Electron microscopy showed the classical double-membrane structure of OSCC EVs. **C** Western blot analysis of several exosomal marker proteins and negative control proteins in OSCC EVs. **D** Confirmation of the hypoxic environment, as detected by the expression of HIF-1α protein. **E** Internalization of EVs derived from OSCC cells (scale bar = 20 μm).

**Additional file 4: Figure S2.** Hypoxic EVs promote radioresistance in OSCC cells **A-B** GW4869 decreased the colony formation of cells treated with H-EV only in irradiated cells (A) but not in non-irradiated cells (B). **C** GW4869 decreased irradiation-induced apoptosis in H-EV-treated OSCC cells. **D** Cell survival curve constructed from colony formation assay data. Cells were treated with GW4869 or DMSO and with EVs derived from normoxic or hypoxic OSCC cells. **E** The number of colonies formed after 8 Gy irradiation was related to H-EV supplementation in a dose-dependent manner (scale bar = 20 μm). **F** The expression of γ-H2AX in irradiated OSCC cells was related to H-EV supplementation in a dose-dependent manner (scale bar = 20 μm). **G** Radioresistance effect of H-EVs on OSCC cells (related to Fig. 1g). **H** GW4869 reversed the radioresistance effect of H-EV on OSCC cells (related to Fig.S2D). D0: mean lethal dose; Dq: quasi-threshold dose; SF2: survival fraction of 2Gy radiation; SER: sensitizing enhancement ratio.

## Data Availability

The datasets used and/or analyzed during the current study are available upon request.

## Abbreviations

OSCC: Oesophageal squamous cell carcinoma
TME: Tumour microenvironment
EVs: Extracellular vesicles
IR: Ionizing radiation
KLF10: Kruppel-like factor 10
PBS: Phosphate-buffered saline
TBST: Tris-buffered saline containing Tween 20
qRT-PCR: Quantitative real-time polymerase chain reaction
RIP: RNA immunoprecipitation
TEM: Transmission electron microscopy
NTA: Nanoparticle tracking analysis
H-EVs: Hypoxic extracellular vesicles
N-EVs: Normoxic extracellular vesicles
H-sh-miR-340-5p-EVs: miR-340-5p knockdown hypoxic extracellular vesicles
H-NC-EVs: Hypoxic negative control extracellular vesicles
UVRAG: UV radiation resistance-associated gene
