# Supplementary material for: Hypoxic tumour cell-derived exosomal miR-340-5p promotes radioresistance of oesophageal squamous cell carcinoma via KLF10
Source: J Exp Clin Cancer Res. 2021 Jan 23;40:38. doi: 10.1186/s13046-021-01834-9 (PMC7825246; doi:10.1186/s13046-021-01834-9)
Supplement: Supplementary file 2 — Additional file 2: Table S2. Clinical characteristics of OSCC patients. [file 13046_2021_1834_MOESM2_ESM.docx]

| Clinical Features | Group |  | Exosomal miR-340-5p expression | | | Histological miR-340-5p expression | | |
| --- | --- | --- | --- | --- | --- | --- | --- | --- |
|  |  | Cases | Low | High | p-value | Low | High | p-value |
| Gender | male | 63 | 30 | 33 | 0.4782 | 35 | 28 | 0.7042 |
|  | female | 25 | 14 | 11 |  | 15 | 10 |  |
| Clinical T stage | cT1+cT2 | 14 | 8 | 6 | 0.5600 | 8 | 6 | 0.9787 |
|  | cT3+cT4 | 74 | 36 | 38 |  | 42 | 32 |  |
| Clinical N stage | cN0 | 30 | 16 | 14 | 0.6529 | 18 | 12 | 0.6647 |
|  | cN1 | 58 | 28 | 30 |  | 32 | 26 |  |
| WHO performance score | 0 | 71 | 34 | 37 | 0.4179 | 40 | 31 | 0.8526 |
|  | 1 | 17 | 10 | 7 |  | 10 | 7 |  |
| Tumor location | Proximal third | 9 | 5 | 4 | 0.9867 | 4 | 5 | 0.7398 |
|  | Middle third | 40 | 20 | 20 |  | 25 | 15 |  |
|  | Distal third | 35 | 17 | 18 |  | 19 | 16 |  |
|  | Oesophagogastric junction | 4 | 2 | 2 |  | 2 | 2 |  |
| Tumor length | ≤5 cm | 37 | 17 | 20 | 0.6661 | 24 | 13 | 0.1943 |
|  | ＞5 cm | 51 | 27 | 28 |  | 26 | 25 |  |
| In-field recurrence in three years | Yes | 32 | 8 | 24 | <0.0001**^*^** | 8 | 24 | <0.0001**^*^** |
|  | No | 56 | 36 | 20 |  | 42 | 14 |  |
| Three-year survival | Yes | 58 | 35 | 23 | 0.0127**^*^** | 39 | 19 | 0.0061**^*^** |
|  | No | 30 | 9 | 21 |  | 11 | 19 |  |

Table S2: Clinical characteristics of OSCC patients
