# Supplementary figures and images for: Hypoxic tumour cell-derived exosomal miR-340-5p promotes radioresistance of oesophageal squamous cell carcinoma via KLF10
Source: J Exp Clin Cancer Res. 2021 Jan 23;40:38. doi: 10.1186/s13046-021-01834-9 (PMC7825246; doi:10.1186/s13046-021-01834-9)

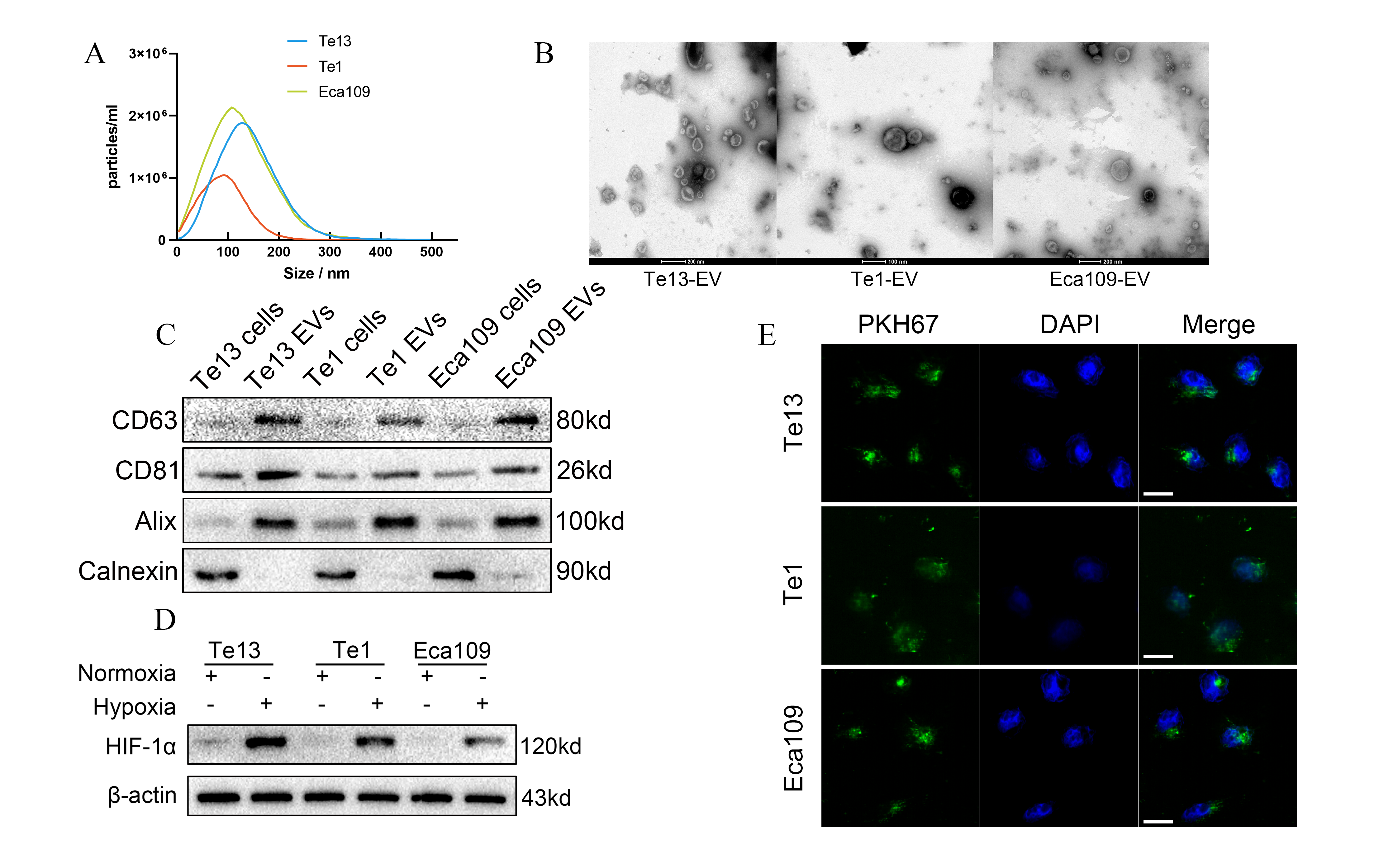

Supplement: Supplementary file 3 — Additional file 3: Figure S1. Characterization of OSCC EVs. A Nanoparticle tracking analysis showing that the diameters of EVs extracted from OSCC cells were mainly between 60 and 180 nm. B Electron microscopy showed the classical double-membrane structure of OSCC EVs. C Western blot analysis of several exosomal marker proteins and negative control proteins in OSCC EVs. D Confirmation of the hypoxic environment, as detected by the expression of HIF-1α protein. E Internalization of EVs derived from OSCC cells (scale bar = 20 μm). [file 13046_2021_1834_MOESM3_ESM.tif]

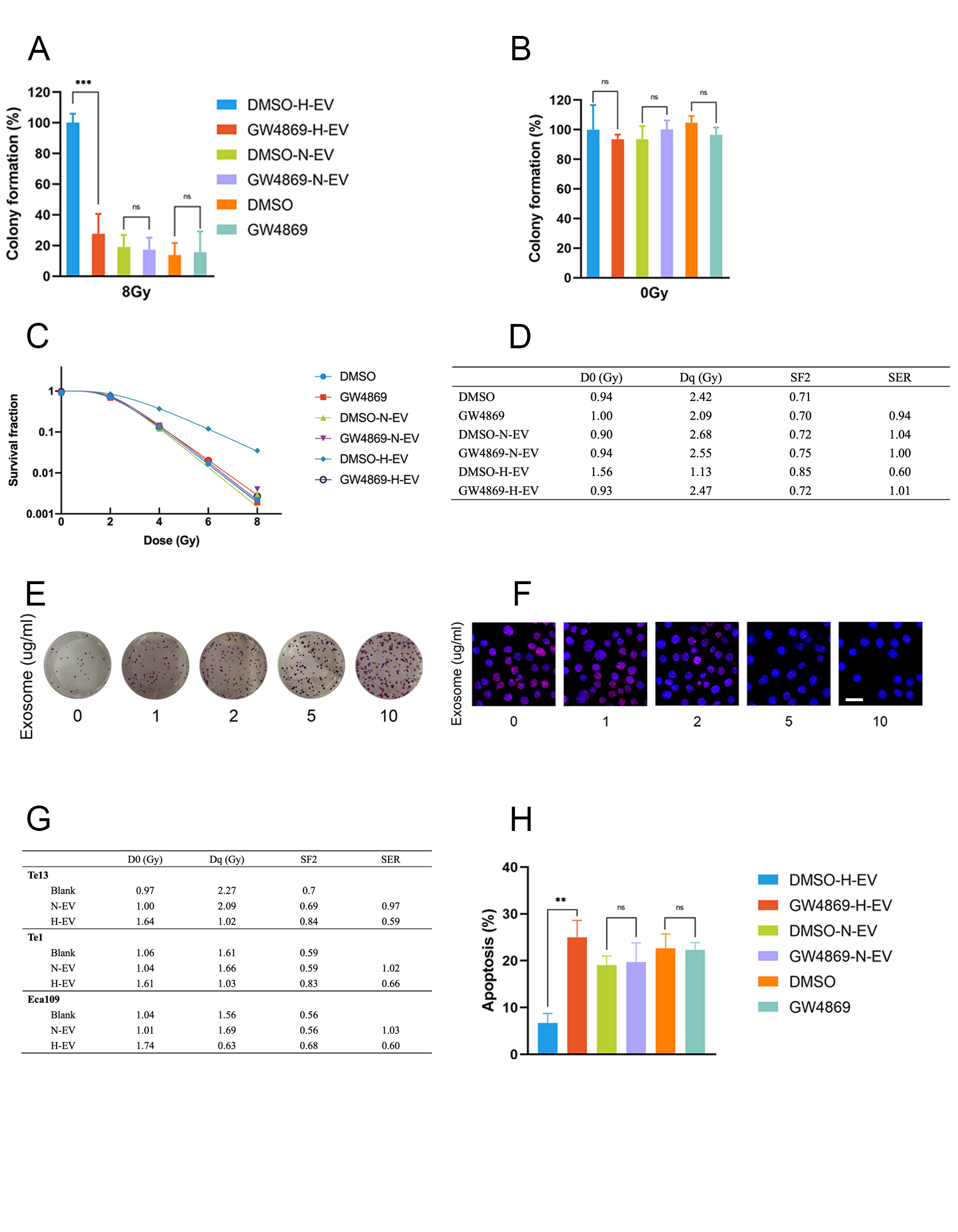

Supplement: Supplementary file 4 — Additional file 4: Figure S2. Hypoxic EVs promote radioresistance in OSCC cells A-B GW4869 decreased the colony formation of cells treated with H-EV only in irradiated cells (A) but not in non-irradiated cells (B). C GW4869 decreased irradiation-induced apoptosis in H-EV-treated OSCC cells. D Cell survival curve constructed from colony formation assay data. Cells were treated with GW4869 or DMSO and with EVs derived from normoxic or hypoxic OSCC cells. E The number of colonies formed after 8 Gy irradiation was related to H-EV supplementation in a dose-dependent manner (scale bar = 20 μm). F The expression of γ-H2AX in irradiated OSCC cells was related to H-EV supplementation in a dose-dependent manner (scale bar = 20 μm). G Radioresistance effect of H-EVs on OSCC cells (related to Fig. 1g). H GW4869 reversed the radioresistance effect of H-EV on OSCC cells (related to Fig.S2D). D0: mean lethal dose; Dq: quasi-threshold dose; SF2: survival fraction of 2Gy radiation; SER: sensitizing enhancement ratio. [file 13046_2021_1834_MOESM4_ESM.tif]
